# 

**Published:** 1891-05-09

**Authors:** 


### NOTICE TO CORRESPONDENTS.

All MS., Letters, Books for Review, and other matters intended for the Editor should be addressed The Editor, The Lodge, Porchester Square, London, W.

The Editor cannot undertake to return rejected M.S., even when accompanied by stamped directed envelope.

**Notice to Advertisers and Subscribers.**

All Advertisements, Orders for Copies of The Hospital, and Business Communications should be addressed to The Publisher, *not to the Editor*, The Hospital, 140, Strand, W.C.

The Cost of Subscription, post free, is as follows:—Per week, 1½d.; per quarter, 1s. 8d.; per half-year, 3s. 3d.; per annum, 6s. 6d. Foreign:—Per annum, 8s. 8d.; payable, in all cases, in advance.

**NOTICE.**—The Index for Vol. IX. (ending March, 1891) is on sale at the Office of this Journal, price 2½d., post free.

### General Charities.

[This Column is devoted to an account of work of charitable institutions other than Medical Charities. The Editor will be glad to receive reports or news of work at 140, Strand. *Philanthropy* should be plainly written on the envelope.]

The eighty-fourth Annual Festival of the **CITY OF LONDON TRUSS SOCIETY** will be celebrated on Tuesday next, the Lord Mayor in the chair. During last year 9,637 poor people of both sexes were relieved by the Society. Subscriptions will be gratefully acknowledged by the Secretary, 35, Finsbury-square, E.C.

The Committee of the London **DIOCESAN COUNCIL FOR THE WELFARE OF YOUNG MEN** were enabled to give a seaside holiday at the Camp, near Deal, to 1,037 boys; this year it is hoped that funds will be forthcoming to send 2,000 boys. 3s. 6d. a-week is required with each lad, who must bear a good character. Offices of the Society: Northumberland Chambers, Charing Cross.

The amount given in relief by the Committee of Management of the **SHIPWRECKED MARINERS' SOCIETY** is a sad testimony to the sorrow and misery brought to many homes by the storms of winter and spring. The relief issue for the Society for the current year already amounts to nearly £8,000. It appears that the total number of sufferers assisted being over 4,000, and the greater part of this has been necessitated by the disastrous March gales. Those who are helped by this institution comprise mariners, fishermen, and boatmen of all grades, and their widows and orphans. Recently several new agencies have been established, among them one at the port of Funchal, Madeira. The annual meeting of the society will take place in June next.

The eighth public dinner of the **ROYAL ALBERT ORPHAN ASYLUM AT SLOUGH** was a success in more ways than one, but particularly as regards the subscription list. The Duke of Clarence and Avondale presided, and pleaded well for the boys and girls now being brought up by the institution, and for those children who, through lack of funds and want of accommodation, are unable to gain admission. Numerous deaths among the old annual subscribers have occurred, whose places badly want filling. A great handicap to the welfare of the Asylum has been the very heavy mortgage, but the late Mr. Junius Morgan, always ready to help a genuine charity, promised one thousand pounds conditionally upon a further sum of £3,000 being raised. Mr. Morgan's executors left the offer open till the evening of the dinner, April 22nd, only, so it was with much anxiety that the new of the amount of the subscription list was awaited. Mr. Tatum announced that £1,500 had been given and subscribed, and a further appeal from Mr. McLean for those present to come to their help that evening brought up the necessary £3,000. Offices, 62, King William Street, E.C.

At the General Court of the **GOVERNESSES' BENEVOLENT INSTITUTION**, which will be held at St. James' Hall Restaurant, 71, Regent Street, on Friday, May 1st, at twelve o'clock, it is proposed to elect ten annuitants, one to the Carrigan annuity of £30, and nine to annuities of £25, and in order to lessen the disappointment of failure, to give £10 to each of the next five on the poll, in addition to the £22 10s. collected by Mrs. Woolloton. This arrangement will not, we fear, tend to lessen the disappointment of those below the lucky number whose merits are quite as great as those of their more fortunate sisters, though they have less influence to bring to bear and less money to spend on canvassing votes. While the voting system exists among our philanthropic institutions, charity is anything but "kind," but gradually, we hope, the cruelty of the system is becoming understood. The objects of the Society, the office of which is at 32, Sackville Street, W., are temporary assistance of governesses in distress, *elective* annuities to aged governesses, which are secured on invested capital; provident annuities, purchased by ladies in any way connected with education, including a savings bank, a home for governesses during the intervals between their engagements, a system of free registration, and an asylum for the aged.

A sum amounting nearly to £2,300 was collected at the festival of the **PROVIDENT CLERKS' BENEVOLENT FUND**, on the 17th instant. The occasion was one of exceptional interest, inasmuch as it was the jubilee of the institution. The fund is one which appeals more particularly to merchants and tradespeople, by whom it is, as it should be, mainly supported. From its foundation it has been liberally assisted by the Bank of England and leading firms in the city, who, as the Lord Mayor very rightly stated, must recognise that commercial success depends very greatly on the accuracy and skill of the clerical work which has to be performed. The objects of the fund are to grant annuities for aged clerks and their widows, and for this purpose a sum of over £90,000 has been expended; to give gratuities; and to grant loans in order to meet cases of temporary difficulty or embarrassment. According to the latest report, there are at the present time 219 annuitants on the fund, in receipt collectively of £4,885 a-year. As the applicants for the sixteen annuities which are to be granted on May 28th next number sixty-seven, it is clear that, if entrusted with more money, the fund can with advantage enlarge its operations, and donations will be gratefully received by the Hon. Secretary, 27, Moorgate Street, E.C.

### SCRAPS AND GLEANINGS.

Baron Hirsch has sent a cheque for £1,000 to the North-West London Hospital.

An old apple woman, who presided at her stall in Piccadilly for many years, has just died at the age of 104.

The Corporation of the City of London has made a grant of one hundred guineas to the Mission to Deep Sea Fishermen.

It has been arranged to form a society of medical officers of health in Scotland, with meeting in Edinburgh, Glasgow, and Perth.

The Merchant Taylors' Company have sent a donation of twenty guineas to the Seaside Camp Fund for London Working Boys.

An entertainment in aid of Cheyne Walk Hospital for Incurable Children was given on May 5th and 6th by Mrs. Henry Wordsworth.

The new premises lately acquired for the Victoria Cottage Hospital Guernsey, were declared open on the 21st ult. by Sir Edgar MacCulloch

The Corporation of the City of London have sent one hundred guineas towards the building fund of the Army Guilds Home for Orphan Daughters of Soldiers.

The Marchioness of Dufferin and Ava opened the three-days' bazaar at Blackheath, in aid of the funds of the Blackheath and Charlton Cottage Hospital.

The freedom of the Plumbers' Company has been presented to Dr. Farquharson, M.P., for the special services rendered by him for many years past to the cause of sanitation.

The Edinburgh Medical Staff Volunteers are to have a camp of instruction within the grounds of Kinross House, the residence of Sir Graham Montgomery, between May 20th and 25th inclusive.

A casket of gold and ivory is to be presented to the Queen by the Corporation of Derby on the occasion of her visit to that town to lay the foundation-stone of the new Derbyshire General Infirmary.

Last Sunday, at St. George's Chapel, Albemarle Street, there was an orchestral service, in which Madame Lilian Nordica took part. The offertory on this occasion was devoted to the Samaritan Hospital.

The Missions to Seamen exhibits in the Howe Gallery of the Royal Naval Exhibition models, &c., of its churches and institutes, &c., illustrating the efforts made to care for sailors at home and abroad.

The Board of Health of the Department of the Seine has appointed Dr. Brouardel, and several members, to represent it, at the International Congress of Hygiene and Demography, to be opened in London on August 10th.

The deaths from influenza in Sheffield have numbered in one week 55, against 15 during the worst week of the epidemic last year. The death rate is said to have reached 57 per 1,000, a rate hitherto unknown in the town.

The village of Rohozora, near Czernovitz, has been the scene of great excitement owing to the appearance of a wolf, who was not killed until it had inflicted serious wounds on 32 persons. The wolf had been suffering from rabies.

The annual report of Eastbourne Homœopathic Convalescent Home states that since the opening of the Home, in August, 1888, not less than 400 persons have been resident, each person having stayed, on an average, three weeks.

A society for the relief of the sick, established in Greenock upwards of a century ago, has just been revived after being dormant for many years, the discovery having been accidentally made that it has a large amount to its credit.

Funds are greatly needed to complete the building of a new church for Plaistow. No help can possibly come from within the parish, as it is entirely poor. The smallest sum will be thankfully received and acknowledged by the Vicar.

An epidemic, whether of cholera or influenza, has not been ascertained, has been raging in Canton. Hundreds of both sexes were falling victims daily, undertakers being unable to turn out sufficient numbers of coffins in which to bury the dead.

Among the latest monthly publications is a kindergarten journal entitled "Child Life," the object of which is to promote union among parents, teachers, and students interested in the philosophic principles and methods of Pestalozzi and Froebel and other prominent educationalists.

The forty-sixth annual report of the Birmingham and Midland Ear and Throat Hospital states that the total number of cases registered at the hospital was 3,994, or 112 less than the number in 1889. Owing to the want of funds the Committee have been reluctantly compelled to reduce the size, and consequently the cost, of the institution.

A clergyman in Brooklyn, N.Y., while giving medicine to a sick child, suddenly laughed, and swallowed the cork of the bottle, which was in his mouth. The cork lodged in the bronchial tube, and although after his removal to the hospital he was placed under ether and tracheotomy performed, the cork could not be removed.

The 114th annual report of the Dumfries and Galloway Royal Infirmary states that 412 in-patients and 3,929 out-patients were under treatment during 1890. The ordinary income was £1,808 0s. 3d., and the expenditure £2,147 18s. 10d., showing a deficiency of £339 18s. 7d., which is, however, only half the amount of the deficit in the preceding year.

### BOOKS RECEIVED.

Baillière, Tindall, and Cox.

"Sea-Sickness, (Cause, Prevention, and Cure)." Thomas Dutton, M.D. Second edition.

Cassell and Co.

"Materia Medica and Therapeutics." J. Mitchell Bruce. Nineteenth thousand.

Kegan Paul, Trench, and Co.

"A Note on the Successful Treatment of Diphtheria; also of the Coryza and Malignant Sore Throat of Scarlatina." Henry Thomas, M.D. Price 1s.

Lambert and Co. (St. Louis, U.S.A.)

"Monographs." By various writers.

Lewis, H. K.

"Elements of Practical Medicine." Alfred H. Carter, M.D. Price 9s.

Percival and Co.

"The Essentials of School Diet." Clement Dukes, M.D. Price 6s.

### The Student of Medicine.

[All communications for this Supplement should be addressed to The Editor of The Hospital, at 140, Strand, with the words, "Students' Column," written in the left hand top corner of the envelope.]

### APPOINTMENTS.

At the Great Northern Central Hospital, Holloway Road, Leonard Remfry, M.A., M.D., B.C. Camb., M.R.C.P., M.R.C.S., has been appointed obstetric physician. At the Metropolitan Hospital, Kingsland Road, C. P. Handson, M.B. Camb., has been appointed assistant house surgeon; C. F. Marshall, M.D., B.S., M.R.C.S., L.S.A., has been appointed house physician; and R. F. Standage, M.R.C.S., L.R.C.P., has been appointed house surgeon. At the Paddington Green Children's Hospital, L. G. Guthrie, M.B. Oxon., M.R.C.P. Lond., has been appointed assistant physician. At the Queen's Hospital, Birmingham, Leonard Parker Gamgee, M.R.C.S., L.R.C.P., has been appointed house surgeon. At the Kent County Asylum, Chartham, near Canterbury, Fred. A. Craig, M.B., has been appointed junior assistant medical officer; William Everett, M.B., has been appointed senior assistant medical officer. At the Erith Cottage Hospital, Leopold J. J. Barnes, M.D., L.R.C.S. Irel., has been appointed medical officer. At the General Infirmary, Leeds, R. L. Daly, M.B., B.S. Durham, has been appointed house surgeon; L. F. West, M.R.C.S., L.R.C.P., has been appointed house surgeon. At King's College, London, Reyton T. B. Beale, F.R.C.S. Eng., has been appointed demonstrator in physiology. At the Kursaal Maloja (Upper Engadine), Michael G. Foster, M.A., M.B. Camb., has been appointed physician.

### VACANCIES.

The following vacancies are announced this week, the amount of salary in each case being given after the name of the institution:

**Resident Medical Officers.**

Birmingham City Asylum, Resident Clinical Assistant—Board, &c. Assistant Medical Officer—Salary £120, &c.
Cancer Hospital (free), Fulham Road, S.W., House Surgeon—Salary £60, &c. Assistant House Surgeon and Registrar—Salary £50, &c.
City of London Lunatic Asylum, Stone, near Dartford, Kent, Clinical Assistant—Board, &c.
Cumberland Infirmary, Carlisle, House Surgeon—Salary £70, &c.
Great Yarmouth Hospital, House Surgeon—Salary £90, &c.
Norwich City Asylum, Hellesdon, near Norwich—Assistant Medical Officer—Salary £100, &c.
Sussex County Hospital, Brighton, Assistant House Surgeon; also House Physician—Salary £60, £80, &c.
Torbay Hospital and Provident Dispensary, Torquay, Junior House Surgeon and Dispenser—Salary £80, &c.

**Non Resident Medical Officers.**

Birmingham and Midland Free Hospital for Sick Children, Steelhouse Lane, Birmingham, Acting Physician—Salary £60.
Boyle Union (Gurteen Dispensary), Medical Officer—Salary £110 and fees.
Cancer Hospital (free), Fulham Road, S.W.—Surgeon, F.R.C.S. Eng., Honorary Pathologist.
Farringdon General Dispensary and Lying-in Charity, 17, Bartlett's Buildings, Holborn, E.C., Honorary Physician.
Queen's College, Birmingham, Professor of Medicine.
Salisbury Infirmary, Dispenser—Salary £105.

### SCHOLARSHIPS AND PRIZES.

**University of Aberdeen.**

List of Students who gained Prizes, Medals, and Certificates in the Faculty of Medicine (*continued*).

MATERIA MEDICA.—Senior Division.

*First Class Certificate and Bronze Medal.*—A. Davidson, Forres, 82 p. c.
*Second Class Certificate.*—F. Beaton, Inverurie, 69.

Junior Division.

*First Class Certificates and Bronze Medals.*—A. Grant, Dufftown, 86 per cent.; D. D. Mackintosh, Aberdeen, 82·5 per cent.; J. Leach, Ardersier, 82 per cent.

*First Class Certificates.*—W. R. Pirie, M.A., Aberdeen, 80·5; G. Savege, Montrose, 80; A. Rajasingham, Ceylon, and J. T. West, Dinnet—equal 79; A. Hogg, M.A., Laurencekirk, 77·5; W. M. Anderson, Alford, 76; W. Ross, Ross-shire, 75·5.

*Second Class Certificates.*—W. Boddie, M.A., Aberdeen, 73·5; J. Sutherland, Bellingham; and A. Brown, Crathie—equal, 73; R. H. Marshall, Aberdeen, 72; C. M. Dawson, Rathen, 71·5, F. B. Graham, Cumberland, 71; W. Trethowan, Victoria, 70; R. E. Kerr, Inverness, 69·5; J. Fraser, Ardersier, 69; W. J. Carmichael, Aberdeen, 67; T. Brander, Garmouth, W. A. G. Russell, M.A., Orkney, and W. Grant, Gartly—equal, 66; J. Wallace, Fearn, 65·5; J. W. Grant, Caithness; and T. H. Galbraith, London—equal, 64; J. Broomhead, Aberdeen, 63·5; G. Geddes, Portgordon, and C. W. Marshall, Greenock—equal, 63; E. M. Griffiths, Wales, 62; A. G. Gall, Aberdeen, 61·5; G. K. Gifford, Shetland, and A. Fraser, Inverness—equal, 61; J. Rust, M.A., Aberdeen, 60·5; R. R. Sutter, New Zealand, and R. Young, South Australia—equal, 60.

*Present at one Examination.*—W. H. de Silva, Ceylon, 75.

### THE STUDENT OF MEDICINE—(*continued*).

WEEKLY ORAL EXAMINATION.

*Special Prize.*—A. Rajasingham.

*High Commendation.*—J. Rust, M.A., A. Grant, W. M. Anderson D. D. Mackintosh, W. R. Pirie, M.A.

PRACTICE OF MEDICINE.—SESSION 1890-91.

SENIOR DIVISION.

*First Class Certificate.*—George W. Tawse, Aberdeen (prize), 94 per cent.

*Second Class Certificates.*—Sydney H. Burnett, Manchester, and James W. de Hoedt, Ceylon—equal, 68 per cent.

JUNIOR DIVISION.

*First Class Certificates.*—John Marnoch, M.A., Aberdeen (prize), 89 per cent.; Alexander Anderson, M.A., Edinkillie, Morayshire (prize), and John Thomson Wilson, Glasgow (prize)—equal, 87; William Findlay, M.A., Aberdeen, 86; Hugh Fraser, M.A., Fearn, Ross-shire, 85; William J. Dewar, Arbroath, 83·5; James Duncan Crow, M.A., Aberdeen, 81; Ernest C. Maguire, Aberdeen, 77·5; Roderick M. MacLennan, M.A., Inverness, 75·5.

*Second Class Certificates.*—William Henry Clark, Woodside, Aberdeen, 74; Thomas Pimley, Preston, Lancashire, 73; William T. Scott, M.A., Fyvie, and James Walker, Orkney—equal, 71·5; Samuel C. Ironside, Aberdeen, 70·5; Alexander G. Allan, M.A., Portsoy, and Patrick W. Diak, M.A., Aberdeen—equal, 68; Ronald C. Macdonald, Aberdeen, 67; Peter R. Ingram, Kildrummy, 66·5; George Mackie, Insch, Aberdeenshire, 65; John H. Lumsden, Fortrose, Inverness-shire, 64; George Wilson, M.A., Strichen, 63·5; George Skeen, Aberdeen, 60·5.

MIDWIFERY.

*Prizemen and First Class Honours.*—John Marnoch, M.A., Aberdeen, 91 per cent.; Wm. Findlay, M.A., Aberdeen, 88; Hugh Fraser, M.A., Fearn, 86.

*First Class Certificates.*—Alexander Anderson, M.A., Edenkillie, 82; Donald Lamond, M.A., Crathie, 79; John Thomson Wilson, Glasgow, 78; George W. H. Tawse, Aberdeen, 75.

*Second Class Certificates.*—William T. Scott, M.A., Fyvie, 73; James Duncan Crow, M.A., and Ernest C. Maguire, Aberdeen—equal, 72; William J. Dewar, Arbroath, 66; George Mackie, Insch, 63; John A. Gilchrist, Banchory, Roderick M. MacLennan, M.A., Inverness, Robert L. Brander, Calcutta, Thomas Pimley, Preston, and Ellerington R. Turner, Sutherlandshire—equal, 60.

SYSTEMATIC PATHOLOGICAL ANATOMY, 1890-91.

*Medallists.*—Ernest C. Maguire and Alexander Anderson, M.A., 97 per cent.; Hugh Fraser, 95 per cent.

*First Class Certificates of Merit.*—John Marnoch, M.A., and G. W. H. Tawse—equal, 93; Patrick Wm. Diack, M.A., 92; Wm. T. Scott, 90; Roderick M. Maclennan, M.A., and J. T. Wilson—equal, 88; Walter W. Sinclair and John H. Lumsden—equal, 83; A. G. Allan, 82; William Findlay and James Duncan Crow—equal, 78; P. R. Ingram and William H. Clark—equal, 77; W. J. Dewar, Thomas Pimley, S. C. Ironside, and Alexander Adams—equal, 75.

*Second Class Certificates of Merit.*—Robert L. Brander, 70; George Mackie, 67; James Bell and J. Leys—equal, 63; Sinclair Couper, 62; Alex. Forbes, 60.

### PASS LISTS.

### Royal Colleges of Physicians and Surgeons in Ireland: Conjoint Scheme.

The following have passed the preliminary examination: J. Barry, J. Bluett, J. Brogan, M. Caraher, M. Carroll, D. Clarke, H. Conyngham, H. Crumplin, W. Cummins, P. Dempsey, L. Fannin, E. Fenton, T. Fisher, P. Fitzsimons, R. Fox, R. Glynn, C. Hodgson, F. Jackson, W. Lawler, J. Leventon, V. Magee, J. Murdoch, J. Murphy, B. McCaul, J. McCarthy, G. McDonnell, D. Spillane, J. Wallace, and A. Goring.

PHYSICS.—J. Benson, M. Carroll, W. Carroll, T. Crean, [illegible]. Crumplin, G. Creighton, W. Darby, P. Dempsey, R. Fox, R. Glynn, A. Halligan, B. Hamilton, M. Hurly, H. Kennedy, L. Lett, V. Magee, J. Maughan, W. McCann, B. McCaul, G. McDonnell, J. Neary, J. O'Keeffe, T. Palmer, J. Pigott, S. Scott, G. Simpson, J. Smyth, W. Stratton, J. Thompson, C. R. Tichborne, F. Wise, and A. Young.

### Durham University.—Faculty of Medicine.

At the second examination for the degree of Batchelor in Medicine, the following candidates satisfied the examiners:

HONOURS (*First Class*): James Atkin Henton White, Queen's College, Birmingham.—*Second Class*: Arthur W. Scott, Yorkshire College, Leeds; Gilbert B. Burrington Kempe, C. Hilary Bryant, Frederick H. Browne, Harold Ernest Gamlen, Stanley McCoull, and Bedlington Howel Morris, College of Medicine, Newcastle-upon-Tyne.

PASS-LIST.—Edward Bromley, Yorkshire College, Leeds; Alfred Glover Cooley; Sheffield School of Medicine; Wm. Henry Daniel Patrick D,Esterre and Harold Darwin Hey, St. Mary's Hospital; Francis W. Fullerton, St. Thomas's Hospital; Thomas Hartigan, Medical School, Catholic University, Dublin; H. Douglas Johns, M.R.C.S., L.R.C.P., Charing Cross Hospital; Geo. Edwyn Middlemist and Morgan Richards, London Hospital; Thos. James Selby, Edinburgh School of Medicine; Frank W. Stokes, University College, London; John Braithwaite, John Stratford Hall, W. Edmund Harker, Arthur Samuel Hedley, Daniel Noel Jackson, Rowland Venables Lloyd-Williams, Henry Bannerman Morison, Alfred Yarker Richardson; Harry Smurthwaite, Arthur E. Thompson, Thomas Christoffel Visser, and Ogden Watson, College of Medicine, Newcastle-upon-Tyne.

At the examination for the Licence in Sanitary Science, the following candidates have satisfied the examiners: Walter H. Cheetham, M.D., Robt. Plenderleith Shearer, M.B., C.M. Glasgow, Thomas Smailes, M.R.C.S., L.R.C.P. Edin., and Chas. Hermann Tattersall, M.R.C.S., L.R.C.P. Lond.

### THE STUDENT OF MEDICINE—(*continued*).

### Royal Colleges of Physicians and Surgeons of Edinburgh and Faculty of Physicians and Surgeons of Glasgow.

The quarterly examinations took place in Edinburgh in April, with the following results:—

First Examination.—Of 31 candidates, the following 21 passed: A. A. Hill, Norwich; A. H. Field, Yorkshire; J. O'Connell, co. Kerry; E. A. Rowan, Kilkenny; S. W. Pitcher, Talbot, Australia; G. H. Williams-Parry, Carnarvon; J. K'Kerrow, Liverpool; G. Prentice, Carnwath; H. W. Vaughan, Torrington; T. P. Powell, Talgarth; C. H. van Straubenzee, Dover; Grace H. Giffen, Roxburghshire; Mary Janet Dodds, Corstorphine; P. Sullivan, co. Cork; W. J. Read, Dublin; J. A. Campbell, Victoria; P. J. Murphy, Cork; W. S. Harvison, New South Wales; Adele Jeanne Chrestien Akyab, India; M. G. Wilkins, Madras; and J. H. Fellows, Wednesbury.

Second Examination.—Of 50 candidates, the following 39 passed: A. W. Hall, Lincolnshire; E. J. Winston-Waters, New Zealand; J. J. Gray, Dublin; T. Messenger, Silloth; W. A. Lloyd-Davies, Abergele; C. A. Smith, Leeds; T. W. Bartlett, Southsea; R. H. F. Bostock, Leicestershire; G. H. Johnstone, Belper; A. Phillips, Coventry; R. J. Blackham, Belfast; R. Morris, co. Cork; F. J. Turnbull, Edinburgh; W. Wardman, Yorkshire; H. A. Jones, co. Cork; H. Campbell, co. Antrim; J. English, Armagh; A. P. Stinson, co. Armagh; P. F. Evans, Cork; Edith Grace Collett, Madras; O. S. Maunsell, co. Waterford; R. D. Jameson, Trinidad; W. R. Thrower, Tasmania; L. C. Murphy, Melbourne: W. L. Lovett, Tullamore, Ireland; J. T. M'Arthur, co. Tyrone; J. Ryan, co. Limerick; P. R. Cairns, Galashiels, A. A. Fermie, India; and A. J. Troughton, Norfolk.

Final Examination.—Of 76 candidates the following 38 passed, and were admitted L.R.C.P. and S.E., and L.F.P. and S.G.: Beatrice M. Harrison, Brighton: W. D. Sweeney, co. Mayo; G. S. Crawford, co. Antrim; J. Orr, co. Antrim; S. Kirkpatrick, Sligo; P. Ukerji, Bombay; C. J. van Coller, Cape Colony; T. J. Davies, Llaurwst; W. Thyne, London; P. Stainsby, Saltaire; V. E. Barrow, Madras; A. A. Macleod, Greenock; J. Reid, Argyllshire; W. Buck, Boston, Lincs.; W. Melville, Bo'ness; J. B. Smith, Montrose; P. R. Crofton, co. Roscommon; D. L. Heggie, Brampton, Canada; L. D. L. Ellis, Manchester; A. B. Masani, Bombay; P. G. Mahoney, Maliopuram; P. J. Hatton, Birkenhead; W. B. Rotheroe, Qweenstown; J. White, Easdale, Argyllshire; A. Dennison, Leeds; R. Johnson, York; W. J. Evans, co. Limerick; Margaret Ida Balfour, Edinburgh; C. R. Martin, Dublin; A. H. Barstow, Spofforth, Harrogate; T. B. Brooke, Cambridgeshire; J. A. Fink, Calcutta; M. B. Gorman, Castletownroche, Cork; J. W. Lewis, Cardiganshire; T. Hamilton, co. Tyrone; J. Barry, Ireland; W. R. W. James, Bangalore; and R. M. Alnes, Rosshire.

### Royal College of Physicians of London.

The following gentlemen have been elected Fellows of the College: William Allen Sturge, M.D. Lond.; Alfred Henry Carter, M.D. Lond.; Richard Grainger Hebb, M.D. Camb.; Sidney Philip Phillips, M.D. Lond.; John James Pringle, M.B. Edin.; William Pasteur, M.D. Lond.; Sidney Harris Cox Martin, M.D. Lond.; Archibald Edward Garrod, M.D. Oxon.; Samuel Herbert Habershon, M.D. Camb.

The following gentlemen have been admitted to the Membership: William Gordon, M.B. Camb.; George Redmayne Murray, M.B. Camb.; Bedford Pierce, M.D. Lond.; Samuel Otway Lewis Potter, M.D. Jefferson Coll.; James Taylor Robb, M.D. Aber.; Gustave Isidore Schorstein, M.B. Oxon.; W. Knowsley Sibley, M.B. Camb.; Ernest Henry Starling, M.D. Lond.; Dawson Fyers Duckworth Turner, M.D. Edin.

### Royal Colleges of Physicians and Surgeons.

The following gentlemen passed the first examination of the Board in elementary anatomy and elementary physiology at the quarterly meeting of the examiners: J. M. Acland, C. Banting, S. Bridger, J. N. Brown, F. J. Coutts, F. G. Crookshank, C. G. Dart, A. G. Ede, P. J. Edmunds, H. P. Emms, A. H. Gerrard, G. H. Goddard, E. G. L. Goffe, A. Hannard, J. C. Hibbert, G. B. Hunt G. P. James, A. W. Jenkins, R. M. Johnston, T. Lambe, J. J. L. Macfarlane, G. V. Miller, W. R. Nattle, G. Phillips, J. E. Phillips, H. Rawsthorne, C. S. Read, J. Richards, W. Roberts, S. E. Shoppee, H. G. F. Stallard, T. A. Starkey, J. W. Stokes, A. T. Thomas, C. F. Wanhill, A. L. A. Webb, C. C. Weeks, S. H. White, and M. Wilkes, students of University College; W. F. Adams, F. R. Baker, E. H. Cooper, R. L. Dickinson, H. E. Dixon, D. E. Evans, C. B. Garman. A. P. Gibbons, A. Gooding, A. S. Grant, C. B. Howse, E. J. Hynes, W. H. Kenrick, A. E. Lovitt, G. B. Mason, W. G. Mortimer, J. H. Power, E. H. Read, J. W. Sames. A. E. Sears, J. L. Sykes, W. P. Thomas, and A. B. Wright, of the London Hospital; W. Allport, J. W. C. Barrett, G. H. E. Bekenn, G. C. Belcher, E. B. Bostock, W. H. Brown, A. Cant, W. M'E. Clendinnin, W. L. Freer, J. Ganner, J. C. Griffiths, J. A. Hall, C. H. Joy, C. Lamplough, F. Laughton-Smith, F. G. Messiter, F. Morris, R. C. Morris, W. P. Nichol, A. W. Nuthall, H. V. Pope, E. P. Satchell, D. L. Thomas, W. G. Thomas, A. X. D. Tomkins, and W. H. G. Wilkes, of Queen's College, Birmingham; A. Amer, R. Armitage, W. N. Barron, A. H. Beadles, H. G. Berry, J. F. Bill, T. B. Bokenham, F. M. Burnett, H. S. Byers, J. S. Chater, A. H. Clarke, F. T. D. Clindenning, A. W. R. Cochrane, M. A. Cooke, G. A. Crace-Calvert, W. F. Cross, J. Currie, S. R. Douglas, D. J. Drake, A. E. Druitt, M. D. Elder, J. R. Evans, W. H. Farmer, J. F. Fernie, E. Folliott, C. S. Foster, E. H. Fryer, G. E. Gardiner, S. Gillies, J. E. Griffith, A. F. Hepworth, A. E. Hodkins, B. W. Holmes, S. C. Hounsfield, B. M. Hughes, J. H. Hugo, H. A. Hutt, A. E. Ireland, M. W. S. Isaacke, R. W. Jameson, D. B. Keown, T. P. Legg, G. J. R. Lowe, T. Martin, J. H. Meacher, G. Miller, A. H. Morris, E. Morris, B. J. L. Owen, A. Pain, M. G. Pearson, W. H. Pope, A. A. Rogers, F. A. Smith, G. H. Sowry, R. D. Stacy, J. S. Stevenson, H. E. Thompson, E. J. Toye, E. P. Turner, and A. Woodward, of St. Bartholomew's Hospital; W. S. Armstrong, D. R. N.

### THE STUDENT OF MEDICINE—(*continued*).

Bernhardt, C. A. A. Coulthard, A. J. Eastcott, R. C. Gayer, G. L. Hay, E. L. Hunt, H. M. Johnson, F. Morley, R. L. Norman, A. E. Rouse, W. A. Sharpin B. B. Tahmisian, C. H. St. M. W. Toke, and C. R. Watson, of St George's Hospital; W. Ashford, W. C. Beddard, P. L. Blaber, T. L. Braidwood, G. W. Brown, P. R. Browning, H. A. Bull, C. L. Chevallier, A. Chopping, A H. Copeman, H. C. Crouch. W. E. Dixon, W. D. Frazer, A. C. Friend, G. G. Genge, P. T. Goodman. A. S. Green, E. Haines, W. N. Heard, W. Herbert, R. A. L Hill, A. L. Home, H. P. Kennard, P. W. Kent, M. H. Laslett, F. G. Layton, H. W. Mills, R. Moreton, E H. T. Nash, K. J. P. Orton, W. H. J. Paterson, L. N. Pentreath, E. L. Perry, G. M. Smith, R. G. Strange, A. C. Swinhoe, and E. O. Thurston, of St. Thomas's Hospital; G. Ashworth, J. T. Barritt, E. W. Battle, A. L. Bostock, J. Broadbent, S. W. Brooke, J. B. Chadwick, W. Chapman, S. Crossley, T. Gregory, J. P. Hall, J. H. Hall, C. G. Higginson, G. R. Howarth, H. F. Jeffery, R Marshall, E. Monks, J. H. Phipps, F. J. Smith, H. Spinks, R. Tarbuck, J. Wallwork, and J. Worthington, of Owens College, Manchester; G. E. Atkins, J. H. Bellamy, A. Cubley, C. R. Dearden, A. M. Edey, F. S. Hardy, H. J. Peel, and W. H. Rowthorn, of the Medical School, Sheffield; W. Badderley, E. W. A. Jeffery, W. H. Lloyd, W. R. Walton, and S. W. Williams, of University College, Liverpool; N. D. Bardswell, H. A. Burridge, J. W. A. Cooper, H. P. Cox, L. C. Dillon, C. C. J. Erhardt, T. A. Hawkesworth, E. E. Head, F. H. Jacob, A. S. M Serley, U. W. N. Miles, J. H. Powell, G. P. U. Prior, J. R. Prior, E. L. M. Rusby, W. M. B. Sparkes, M. M. Townsend, and P. C. E. Tribe, of King's College; C. Beven, C. M. Beadnell. J. W. Bird, F. S. Brereton, W. F. Byford, W. A. Carden, E. Coleman, J. J. Coleman, S. Copley, L. Cotterill, W. L. B. Davies, C. A. Ensor, C. H. Fagge, E. Fisk, C. J. Francis, T. H. Green, E. S. Hall, W. S. Handley, C. J. Harnett, R. Hewlett-Hayes, J. H. Horton, B. Instone, M. P. Jones, C. J. Kearney, F. J. Kenyon, J. G. O. H. Lane, H. Leader, C. R. Lucas, A. J. Makepeace, H. A. Moffat, J. Moore, H. D. Packer, B. F. Pendred, A. E. Philipps, B. G. Reynolds, B. A. Richmond, R. L. Roberts, J. Robertson, W. J. Rowland, F. W. Rowland, A. Salter, E. T. Scowby, E. T. Shorland, H. J. M. Smith, H. J. Spon, F. J. Steward, H. B. Stoner, D. W. Thomas, E. R. Thomas, T. M. Thomas, E. H. Tipper, P. E. Tresidder, E. H. Van Someren, P. N. Vellacott, and L. Worts, of Guy's Hospital; S. B. Blomfield, F. E. Clay, F. Copeland, A. H. Evans, G. F. S. Genge, S. A. M. Gilbert, G. C. Hancock, H. Morris, R. J. Stillwell, J. M. G. Swainson, and S. G. Tippett, of Westminster Hospital; T. W. W. Bovey, E. A. Dorrell, C. R. Dykes, D. S. Gerrish, G. F. Gregory, G. J. Hare, E. W. Ormerod, A. E. H. Pinch, and W. M. Willis, of the Medical School, Bristol; W. P. Brookes, G. B. Clarke, E. T. Clayton, W. R. Coldicot, J. H. L. Duke, E. H. Fricke, J. C. Furness, D. P. Gabell, W. Hardcastle, W. C. D. Hills, D. W. Jones, J. W. Leake, W. J. Pike, M. P. Powell, H. S. Prideaux, S. T. Reid, H. J. Stevens, D. W. Wiseman, W. W. Wolliscroft, W. E. Wyborn, O. C. Yonell-Carter, and C. W. Young, of Charing Cross Hospital; L. H. Callender, J. E. P. Davies, J. L. Davies, J. Glover-Heath, R. J. E. Hanson, W. K. Hay-Coghlin, W. St. G. Hill, S. P. James. H. G. Jones, J. N. Macdonald, C. Matthews, E. G. Mayo, H. T. L. Roberts, E. R. Rost, O. Shields, J. C Smellie, P. M. Smith, W. W. Walker, and W. J. Woodman, of St. Mary's Hospital; H. M. Case, A. P. Coker, G. W. Connor, E. J. Dobbin, F. L. Dodd, L. A. Fawssett, J. Gardener, A. C. Greenwood, T. T. Harratt, T. Herbert, J. J. Hughes, A. S. Lawrence, F. R. Mann, R. J. Mayne, A. P. Parker, H. Raper, C. H. J. Robinson, A. H. G. Rose, L. Savin, A. G. L. Smith, M. K. Tebay, N. E. Thomas, A. Wells, and J. H. Wilson, of Middlesex Hospital; W. Cooper, A. P. Cummings, A. M. Dow, C. W. Eames, T. S. Hartley, W. E. Heal, A. E Hutton, A. E. Little, E. J. Lumb, C. A. Phillips, E. H. Phillips, W. F. Rawson. W. A. H. Waite, and J. H. Wigham, of Yorkshire College, Leeds; E. W. Ormerod, of Cambridge and St. Bartholomew's Hospital; M. H. C. Palmer, of Cambridge and London Hospital; and W. M. Whitehouse, of the University of Durham.

The following gentlemen passed in anatomy only: Messrs. W. Barton, F. Hewitt, and K. R. D. Shaw, of Owens College, Manchester; C. Basan, H. L. Evans, L. Lloyd, H. B. Oldham, and A. B. R. Sworn, of University College; W. A. Bramsdon, W. H. Crossley, and D. L. Jones, of St. Bartholomew's Hospital; H. J. Clague, J. G. Holmes, and G. W. White, of the Medical School, Sheffield; P. S. Crookes and J. H. De Villiers, of St. Thomas's Hospital; R. F. Clark, R. B. Stamford, and E. A. Taylor, of Guy's Hospital; E. E. Crowther and G. Y. Myrtle, of Yorkshire College, Leeds; E. E. Ellery, R. H. Mornement, M. B. Pinchard, H. L. Porteous, and J. M. Wilson, of Middlesex Hospital; E. M. B. Payne and J. P. Prell, of London Hospital; O. F. Pritchard, of King's College; and P. Wykesmith, of St. George's Hospital.

The following gentlemen passed in physiology only:—Messrs. J. A. Alston, J. Blackwood, and J. H. Cook, of University College; J. A. Angus, S. G. Harding, and A. F. Palmer, of Middlesex Hospital; T. H. Bailey, J. R. Benson, R. G. Knox, and G. U. Smith, of King's College; S. S. Broadbent, of the Medical School, Sheffield; R. W. S. Christmas, E. H. S. R. Greene, R. E. Harrison, E. C. Montgomery, and E. Moseley, of Charing Cross Hospital; W. L. Cockcroft, C. Coventry, E. Fielden, A. Hodge, R. S. Thorpe, H. J. Watts, and A. Whitfield, of Owens College, Manchester; J. L. Elliott, P. V. Fry, P. G. Lodge, and P. Macaulay, of Yorkshire College, Leeds; J. Gundlach, G. C. W. Pimbury, and E. Pratt, of St. Bartholomew's Hospital; F. J. H. Cann, J. Hora, F. Lond, J. R. Steinhaeuser, M. E. Tresidder, T. J. Vick, and W. E. Waymark, of Guy's Hospital; K. M. Johnson, G. S. Thomson, and A. W. D. Thomson, of St. George's Hospital; A. T. Jones, E. L. Roberts, and G. R. Sparrow, of University College, Liverpool; J. R. Kingdon, of Cambridge University; R. A. MacLeod, and A. E. Rutherford, of Westminster Hospital; F. W. Phillips and J. B. Wall, of St. Mary's Hospital; and G. A. Leon, of London Hospital.

